# Singlet–Triplet Gap in Covalently and Weakly Bound Carbenes: Studying the Dependence on the Exchange–Correlation Functional

**DOI:** 10.1021/acsomega.5c07611

**Published:** 2025-10-17

**Authors:** Pablo Maiz-Pastor, Éric Brémond, Ángel José Pérez-Jiménez, Carlo Adamo, Juan Carlos Sancho-García

**Affiliations:** † Department of Physical Chemistry, University of Alicante, E-03080 Alicante, Spain; ‡ Université de Paris, ITODYS, CNRS, F-75006 Paris, France; § Chimie ParisTech, PSL Research University, CNRS, Institute of Chemistry for Life and Health Sciences (i-CLeHS), FRE 2027, F-75005 Paris, France

## Abstract

We systematically analyze here the performance of density functional approximations for the calculation of the singlet–triplet gap of covalently bound carbenes (i.e., substituted arylcarbenes of increasing size and with different electroactive substituents) and weakly bound carbenes (diphenylcarbene noncovalently interacting with H_2_O, CH_3_OH, ClCF_3_, BrCF_3_, and ICF_3_ molecules). The calculations revealed an unexpected functional dependence, in the sense that low levels of the functional hierarchy (e.g., BLYP or PBE) provide lower errors than more sophisticated functionals (e.g., hybrid and double-hybrid expressions), thus evidencing a subtle yet marked interplay between the ingredients of any modern exchange–correlation functional. The decomposition of the results as a function of those ingredients allowed us to isolate those functionals overestimating (by default) the energy stability of the triplet state and how (nonempirical) formulations of double-hybrid functional are able to cope with that initial bias. We recommend double-hybrid functionals (e.g., PBE-QIDH) to get robust and sufficiently accurate results (averaged deviation of ±1–2 kcal/mol with respect to reference results) for other applications and studies involving these challenging carbene species.

## Graphical abstract

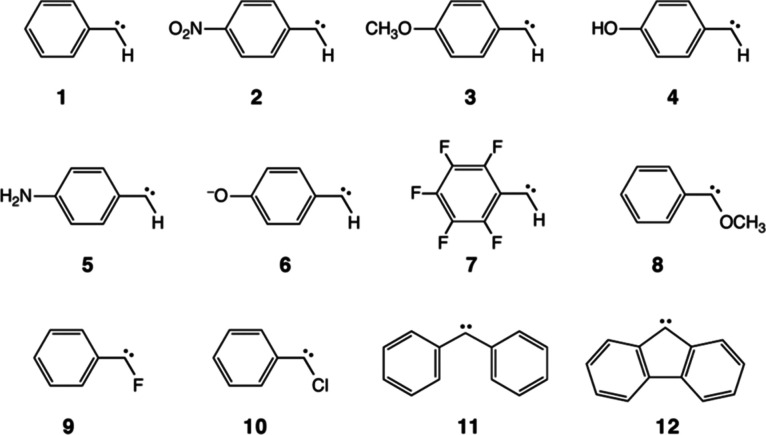

## 1 Introduction

Carbenes are divalent compounds, and thus highly reactive and short-lived species, of general and simplified formula R–(C)–R′ or R=C:, which emphasizes the presence of two unshared (spin-paired or spin-unpaired) electrons on a C atom.^1^ They play a key role in synthetic organic chemistry, or act as intermediate species in reaction mechanisms, and thus constitute a versatile and interesting chemical platform.^2–5^ Although one would initially expect a triplet ground-state for carbenes, arising from the spin-unpairing, the energy difference between the singlet (*S* = 0) and triplet (*S* = 1) electronic states of these systems is known to strongly vary depending on many factors, such as substitution (inductive and mesomeric) effects^2,6^ (e.g., σ-electron-withdrawing substituents favor the singlet vs the triplet state) as well as geometrical factors (e.g., steric interactions protecting the highly reactive carbene center and/or spin delocalization by extending the conjugation core would help to stabilize the triplet state). Additionally, although from the 1970s onward finding molecules that violate Hund’s rules was actively tackled by theoretical methods, there is a recent and increasing interest in organic systems displaying Hund’s rule violation, for both ground and excited states, with lessons gained along the year also pointing to a marked dependence on the exchange–correlation functional used.^7,8^ Therefore, the accurate knowledge of the singlet–triplet energy gaps of real-world carbenes is of high importance for the chemistry of these compounds,^9^ which is however challenging to predict due to all the concurring (stereo)­electronic effects mediating the gap, thus needing to be cost-efficiently captured by quantum-chemical methods. Actually, as it has been widely documented in the literature before,^10–17^ one is forced to go beyond low- or medium-cost methods for accurate predictions and/or for finding an agreement between theoretical and experimental results for carbenes, but at the price of a high (and often unaffordable) computational cost due to the unfavorable scaling with system size of most of the correlated methods so far applied.

Therefore, aiming at shedding light on the performance of modern theoretical methods for this challenging issue, we will focus here on a set of recently studied arylcarbenes, the AC12 data set,^18^ as it is often done in recent times for the benchmarking of quantum-chemical methods, see Figure 1. The data set comprises pristine (**1**) and substituted phenylcarbene (**2–10**) as well as diphenylcarbene (DPC) (**11**) and fluorenylidene (**12**), for which nearly exact (gas-phase) reference values are available thanks to the original work^18^ of Neese et al. In that study designing the AC12 data set, besides providing nearly exact reference results for that singlet–triplet energy difference, it was already shown that Hartree–Fock and second-order Møller–Plesset perturbation theories (MP2), scaling respectively as $O\left(N^{4}\right)$ – $O\left(N^{5}\right)$ with *N* related to the system size, were unable to deliver accurate results unless a costly orbital-optimization procedure is done for the latter (OO-MP2 ^19^). Actually, those OO-MP2 calculations were also shown^20^ to deliver accurate structures compared to reference results. An alternative based on a linearized open-shell MP2 (RO-LMP2) approach was also successfully applied^21^ to the AC12 data set as well as a new self-consistent perturbation theory named one-body MP2 (OBMP2)^22^ or a multireference CASPT2 extension coupled with solvent effects,^23^ showing the constant interest in developing, benchmarking, and implementing theoretical methods for these challenging systems.

**Figure 1:**
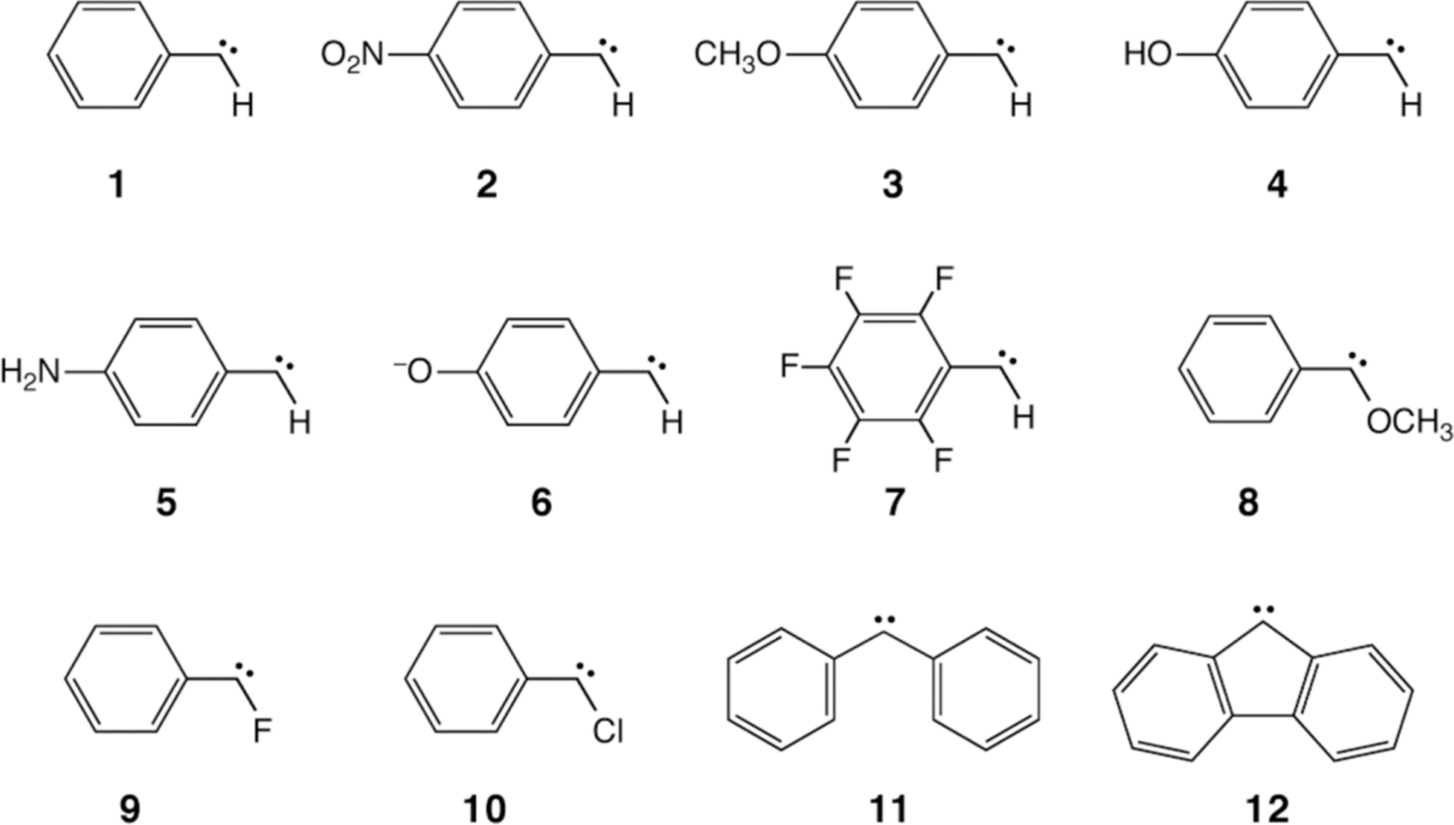
Chemical structures of the molecules **1–12** (AC12 data set). The H atoms of benzene(s) are omitted for clarity.

Obviously, the use of density functional theory (DFT) might constitute an excellent alternative to those wave function-based methods mentioned above, although historical DFT applications to carbenes showed some inconsistent performance in the past.^24,25^ However, DFT has experienced a blooming in the last decades with more accurate and robust expressions, including (but not only) hybrid, range-separated, and double-hybrid functionals. Interestingly, the widely used set of PBE, BLYP, PBE0, or B3LYP functionals were also applied before^18,26^ to the AC12 data set, with a surprising deterioration of the results going from BLYP to PBE and from B3LYP to PBE0. Actually, the performance of these and other functionals was found to strongly vary for these systems, spanning a considerably large error range of 1–15 kcal/mol and showing some erratic behavior across the hierarchy of DFT methods. Successful alternatives might be local-hybrid functionals^27,28^ as well as double-hybrid density functionals^29–31^ although only two (semiempirical) expressions of the latter class (i.e., B2-PLYP^32^ and PWPB95 ^33^) were employed so far. Note that the latter methods also scale as $O\left(N^{5}\right)$ but were found to be much more accurate than MP2 in their preliminary application to the AC12 data set.

Furthermore, it is also known that hydrogen-bonded or halogen-bonded complexes, where the carbene acts as electron donor, can modulate the singlet–triplet energy difference or switch the electronic ground state from triplet to singlet.^34,35^ Additionally to the study of the AC12 data set, we will complement it by tackling weakly (intermolecularly) bound carbene systems based on DPC (compound **11** of the AC12 data set) and H_2_O and CH_3_OH as hydrogen-bond donors, and MCF_3_ molecules (M = Cl, Br, I) as halogen-bond donors, for which reference results are also available in the literature^36^ at the same wave function-based level than that used for the AC12 data set.

Hence, seeing the results previously obtained for covalently and weakly bound carbenes,^18,26,36^ but applying a limited set of DFT methods, we would like to shed light on the performance of semiempirical and nonempirical models along the hierarchy of semilocal, hybrid, and double-hybrid functionals as well as isolate the underlying reasons for their performance. Furthermore, we have developed and systematically assessed along the last years a set of nonempirical double-hybrid functionals (i.e., PBE0-DH^37^ and PBE-QIDH^38^ as well as some variants) built upon a set of exact mathematical conditions and physical constraints, and thus not depending on any parametrization or training data sets.^39^ They have also been successfully applied before^40,41^ to spin-state splitting energies of organometallic compounds (e.g., complexes of Fe and Co in different oxidation states coupled with diverse ligands) and their validation will be thus also extended here to these arylcarbenes and weakly bound carbenes. Note that the truly nonempirical nature of the functionals selected here, not involving any parametrization in their development, will allow us to analyze and disentangle the energy contributions driving the results.

## 2 Computational Methods

### 2.1 Choice of DFT Functionals

The definition of the functionals selected here is based on the following general form, which easily defines any exchange–correlation density functional (*E*_*xc*_[ρ]) belonging to any family of the employed expressions

1 $$E_{xc}\left[\rho \right]=a_{x}E_{x}^{EXX}+\left(1-a_{x}\right)E_{x}\left[\rho \right]+a_{c}E_{c}^{PT2}+\left(1-a_{c}\right)E_{c}\left[\rho \right]$$

where *E*_*x*_^EXX^ represents the EXact-eXchange (EXX) exchange, weighted by the coefficient *a*_*x*_, and *E*_*c*_^PT2^ represents the correlation energy obtained at the second-order perturbation theory (PT2) weighted by the coefficient *a*_*c*_ and *E*_*x*_[ρ] and *E*_*c*_[ρ] are, respectively, the exchange and correlation density functionals chosen. The semilocal functionals are simply obtained after setting *a*_*x*_ = *a*_*c*_ = 0. A hybrid functional is by definition defined as that holding *a*_*x*_ ≠ 0 and *a*_*c*_ = 0, whereas a double-hybrid functional is obtained if *a*_*c*_ ≠ 0. Following the same line of reasoning when deriving nonempirical hybrid and double-hybrid models, the PBE^42^ and r^2^SCAN^43^ exchange and correlation functionals will be chosen for *E*_*x*_[ρ] and *E*_*c*_[ρ]. Recall that PBE and r^2^SCAN are some of the most representative nonparameterized expressions belonging to the generalized gradient approximation (GGA) and meta-GGA approximations, respectively.

Table 1 summarizes the values of the *a*_*x*_ and *a*_*c*_ coefficients for each of the functionals selected here. If one sets *a*_*x*_ = 1/4 and *a*_*c*_ = 0, we obtain the hybrid (and commonly used) PBE0 ^44^ model, or r^2^SCAN0 if the r^2^SCAN expression for *E*_*xc*_[ρ] is used instead, while the double-hybrid family of functionals is recovered by setting *a*_*x*_ ≠ 0 and *a*_*c*_ ≠ 0 as it is done for the PBE0-DH^37^ (*a*_*x*_ = 1/2 and *a*_*c*_ = 1/8), PBE-QIDH^38^ or r^2^SCAN-QIDH^45^ (*a*_*x*_ = 3^–1/3^ and *a*_*c*_ = 1/3) functionals. For all this set of functionals, a correction for (intra- and intermolecular) noncovalent dispersion effects can also be added through the modern D4 scheme,^46^ which is also available for all the expressions considered here.^47–49^ For comparison purposes, we also consider the widely used BLYP-based family of functionals: BLYP,^50,51^ B3LYP,^52^ B2-PLYP^32^ (*a*_*x*_ = 0.53 and *a*_*c*_ = 0.27), and B2GP-PLYP^53^ (*a*_*x*_ = 0.65 and *a*_*c*_ = 0.36) with and without the corresponding D4 correction.

**Table 1:** Values of *a*_*x*_ and *a*_*c*_ for the Density Functionals Used Here

functional	*a*_*x*_	*a*_*c*_
BLYP	0.0	0.0
B3LYP	0.2	0.0
B2-PLYP	0.53	0.27
B2GP-PLYP	0.65	0.36
PBE	0.0	0.0
PBE0	1/4	0.0
PBE0-DH	1/2	1/8
PBE-QIDH	3^–1/3^	1/3
r^2^SCAN	0.0	0.0
r^2^SCAN0	1/4	0.0
r^2^SCAN-QIDH	3^–1/3^	1/3

### 2.2 Computational Details

The singlet–triplet (adiabatic) energy difference is calculated for all of the systems as Δ*E*_ST_ = *E*(T//T) – *E*(S//S), with restricted or unrestricted calculations fixed for singlet (S) and triplet (T) states, respectively. The geometry of all the compounds of the AC12 data set was taken from ref 18 (optimized at the B3LYP-D3­(BJ)/def2-TZVPP level) and those of the weakly bound systems from ref 36 (optimized at the OO-SCS-MP2/def2-TZVPP level), as well as the corresponding reference values obtained at the coupled-cluster single, double, and perturbatively estimated triples at the complete basis set limit, or CCSD­(T)/CBS, for the singlet–triplet energy difference Δ*E*_ST_, whose high cost was alleviated by employing the domain-based pair natural orbital (DLPNO) technique for the weakly bound systems. In such a way, we avoid geometry-dependent effects and can readily compare both sets of reference and DFT-based results for both covalently and weakly bound carbenes.

The very large def2-QZVPPD and def2-QZVPP basis sets will be used for AC12 and the weakly bound systems, respectively. The possible influence of the basis set size on the results (for the AC12 data set) will be complementarily assessed for double-hybrid methods, employing the hierarchy of increasingly larger def2-TZVP, def2-TZVPP, def2-TZVPD, and def2-QZVPPD basis sets, with the latter already providing results close enough to the basis set limit.^54^ All the calculations are done with finer-than-default grids and under the RIJCOSX approximation^55^ (together with the corresponding auxiliary def2/JK and def2/C basis sets) as implemented in the ORCA 5.0 package^56^ used for all the calculations reported here.

The assessment of the methods will be done by inspecting the “mean-signed deviation” (MSD, defined as $MSD=\frac{1}{n}\sum_{i}^{n}x_{i}$ ), the “mean absolute deviation” (MAD, defined as $MAD=\frac{1}{n}\sum_{i}^{n}\left|x_{i}\right|$ ), the “root mean-squared deviation” (RMSD, defined as $RMSD=\sqrt{\frac{1}{n}\sum_{i}^{n}x_{i}^{2}}$ ), and the maximum (unsigned) error (MAX, defined as $\mathrm{MAX}=\max\left(|x_{i}|\right)$ , *∀i* ∈ (1, ..., *n*)), being *n* the number of systems considered (*n* = 12 for the AC12 data set and *n* = 5 for the set of weakly bound complexes) and *x*_*i*_ the difference between the DFT-calculated and the reference Δ*E*_ST_ values.

## 3 Results and Discussion

### 3.1 Global Performance of Functionals on the AC12 Data Set

The sign of the reference Δ*E*_ST_ values, taken from ref 18, already determines if the singlet (Δ*E*_ST_ > 0) or triplet (Δ*E*_ST_ < 0) is the energetically favored spin state, with Δ*E*_ST_ > 0 calculated for compounds **5**, **6**, **8**–**10** of the AC12 data set with reference values (in kcal/mol) of 1.35, 22.54, 25.05, 17.05, and 7.65, respectively. For the rest of the compounds, the negative reference values of Δ*E*_ST_ oscillate between the lowest (−0.08 kcal/mol) for compound **3**, followed by −0.30 kcal/mol for compound **4**, to the highest (−7.89 kcal/mol) for compound **2**. Therefore, one can see that the reference values are comprised roughly speaking between −8 and 25 kcal/mol, but with a pair of them with an easily reversible sign showing their low value (|Δ*E*_ST_| < 0.3 kcal/mol), which might strongly depend on the accuracy and robustness of the computational model in use. All the reference values can be found in the Supporting Information. Table 2 consequently presents the error metrics (MSD, MAD, RMSD, and MAX) for the set of functionals used here, with and without the D4 dispersion correction. We will separately analyze the performance of the BLYP-based family of functionals (i.e., BLYP, B3LYP, B2-PLYP, and B2GP-PLYP) and the PBE-based (i.e., PBE, PBE0, PBE0-DH, and PBE-QIDH) and r^2^SCAN-based (i.e., r^2^SCAN, r^2^SCAN0, and r^2^SCAN-QIDH) family of functionals as well as the influence of the basis sets size.

#### 3.1.1 The BLYP-Based Family of Functionals

Analyzing first the set of BLYP-based functionals, the performance of BLYP and B2-PLYP methods is remarkably accurate, providing MAD values as low as 1.0–1.2 kcal/mol, closely followed by B3LYP and B2GP-PLYP with MAD values of 2.2–2.3 kcal/mol. Comparing the results by double-hybrid functionals between them, one can see a deterioration of the error metrics by more than 1 kcal/mol going from B2-PLYP to B2GP-PLYP. Note that the only difference between B2-PLYP and B2GP-PLYP is a larger fraction of both *a*_*x*_ and *a*_*c*_ coefficients for the latter, see eq 1 and Table 1. We also note (see the Supporting Information) that both B2-PLYP and B2GP-PLYP predicted the wrong sign of Δ*E*_ST_ for compounds **3** and **4**, with errors as high as 1.5–2.5 kcal/mol for these two systems. Perusing now the difference between pristine and D4-corrected error metrics, one can easily see how the D4 correction modifies the MAD marginally by ± 0.1 kcal/mol, although improving it only for B3LYP and not for the other functionals BLYP, B2-PLYP, or B2GP-PLYP. As it was also expected, the effect of that correction was the lowest among the BLYP-based functionals for the double-hybrid B2-PLYP and B2GP-PLYP models, due to the (partial) inclusion of the correlation energy by the *E*_*c*_^PT2^ term.

#### 3.1.2 The PBE-Based Family of Functionals

The performance of the nonempirical PBE-based functionals is also gathered in Table 2, with PBE providing slightly lower errors than the PBE0 (hybrid) or PBE0-DH (double-hybrid) models. That relative (and unusual) performance mimics that found before for the BLYP-based hierarchy of functionals, with BLYP also providing lower errors than the B3LYP (hybrid) or B2GP-PLYP (double-hybrid) models. Overall, these PBE-based functionals gave higher errors than the corresponding BLYP-based functionals belonging to the same rung of models (i.e., compare BLYP with PBE or B3LYP with PBE0). As another proof-of-concept, we have also used the semilocal TCA functional^57^ through the libxc library,^58^ to find a MAD value of 3.1 kcal/mol and thus comprised between that of BLYP and PBE, indicating again the marked dependence on the results on the exchange–correlation functional used. The performance of PBE-QIDH is, however, considerably more accurate than the rest of the PBE-based models, halving the error metrics with respect to the best of the others and providing a low and competitive MAD of 2.0 kcal/mol. Interestingly, PBE-QIDH was able to predict the correct Δ*E*_ST_ sign for all of the AC12 members, which is not the case for any other double-hybrid functional tested here. The use of a range-separated version of the PBE-QIDH double-hybrid functional, the nonempirical RSX-PBE-QIDH^59^ model with ω = 0.27 bohr^–1^, did not bring any advantage and it actually increases the MAD by around 1.5 kcal/mol. That increase is reminiscent of what was already reported in the literature^26^ for the CAM-B3LYP model, a range-separated version of the B3LYP functional, which also increased the error by more than 1 kcal/mol. The addition of the D4 correction for dispersion had almost a negligible effect for the PBE or PBE0 models (reducing the, for example, MAD by less than 0.05 kcal/mol) and left the PBE0-DH and PBE-QIDH error metrics almost unaffected. Additionally, we notice that increasing both the *a*_*x*_ and *a*_*c*_ weights from PBE0-DH (*a*_*x*_ = 3^–1/3^ and *a*_*c*_ = 1/3) to PBE-QIDH (*a*_*x*_ = 1/2 and *a*_*c*_ = 1/8 for PBE0-DH) largely improved the results, contrarily to what happened before comparing B2GP-PLYP and B2-PLYP.

**Table 2:** MSD, MAD, RMSD, and MAX Errors (kcal/mol) for the AC12 Dataset and All the Functionals Investigated^a^

functional	MSD	MAD	RMSD	MAX
BLYP	–0.67	1.16	1.51	3.38
BLYP-D4	–0.52	1.27	1.58	3.73
B3LYP	–2.19	2.19	2.41	3.86
B3LYP-D4	–2.09	2.09	2.34	4.06
B2-PLYP	0.91	1.05	1.31	2.85
B2-PLYP-D4	0.96	1.10	1.40	3.24
B2GP-PLYP	2.19	2.26	2.56	5.64
B2GP-PLYP-D4	2.22	2.28	2.61	5.89
PBE	–4.19	4.19	4.40	6.15
PBE-D4	–4.12	4.12	4.36	6.20
PBE0	–6.55	6.55	6.60	7.70
PBE0-D4	–6.49	6.49	6.55	7.67
PBE0-DH	–5.87	5.87	5.91	7.09
PBE0-DH-D4	–5.85	5.85	5.90	7.08
PBE-QIDH	–1.72	2.05	2.16	3.61
PBE-QIDH-D4	–1.70	2.06	2.17	3.60
RSX-PBE-QIDH	–3.43	3.43	3.62	4.82
r^2^SCAN	–9.33	9.33	9.40	11.02
r^2^SCAN-D4	–9.31	9.31	9.39	11.01
r^2^SCAN0	–11.61	11.61	11.64	13.17
r^2^SCAN0-D4	–11.59	11.59	11.62	13.16
r^2^SCAN-QIDH	–5.33	5.33	5.45	6.65
r^2^SCAN-QIDH-D4	–5.33	5.33	5.45	6.65

a All calculations are done with the def2-QZVPPD basis set.

#### 3.1.3 The r^2^SCAN-Based Family of Functionals

Furthermore, Table 2 also shows the results for the r^2^SCAN-based functionals, which perform significantly worse than their PBE-based counterparts (i.e., compare PBE with r^2^SCAN, PBE0 with r^2^SCAN0, or r^2^SCAN-QIDH with PBE-QIDH) with the dispersion correction neither affecting the results, very likely due to the inclusion of medium-range electronic effects by the r^2^SCAN exchange and correlation functionals by themselves. Actually, we have also shown that the performance of r^2^SCAN for large data sets (e.g., GMTKN55, S66 × 8, O24 × 5, etc.) resembles more that of a hybrid functional than that of a semilocal functional,^45^ and thus the inclusion of a portion of exact-like exchange (*a*_*x*_ = 1/4 for r^2^SCAN0) can deteriorate the results even further. These results agree with those found before using a spin-scaled version of r^2^SCAN-based double-hybrid functionals^60^ where the MAD of the corresponding (spin-scaled) r^2^SCAN0-DH version was as high as 9.56 kcal/mol, thus indicating that the introduction of a higher exact-like exchange weight (*a*_*x*_ = 1/2 for r^2^SCAN0-DH) does not work in the right direction. However, a much improved performance is found for the r^2^SCAN-QIDH double-hybrid version, approaching the performance of PBE0-DH but still being less accurate than the corresponding PBE-QIDH functional, even if its exact-like exchange weight is now higher (*a*_*x*_ = 3^–1/3^ for r^2^SCAN-QIDH).

#### 3.1.4 The Influence of the Basis Set Size

The influence of the basis sets size is analyzed next in Table 3 for the set of PBE-QIDH­(-D4) and r^2^SCAN-QIDH­(-D4) models, seeing their dependence with a relatively high value of *a*_*c*_ on the *E*_*c*_^PT2^ correlation energy, which is known to converge more slowly with the basis set size than the rest of the energy terms of eq 1. We can observe that all the error metrics (MSD, MAD, RMSD, and MAX) consistently decrease upon extending the basis set, with a more marked decrease of around 0.2–0.3 kcal/mol when going from a triple-ξ (def2-TZVPP) to a quadruple-ξ (def2-QZVPP) than when adding additional polarization (def2-TZVPP) and diffuse (def2-TZVPPD) functions to the triple-ξ basis set. However, a reversed trend, and thus possibly spurious performance, is observed with B2-PLYP, for which the error metrics increase from the def2-TZVP to the def2-QZVPPD, after systematically stabilizing the singlet state by 0.2–0.4 kcal/mol in all cases, indicating that previously published values with this method (and the def2-TZVPP) might not be sufficiently converged with respect to the basis set size.

**Table 3:** MSD, MAD, RMSD, and MAX Errors (kcal/mol) for the AC12 Dataset with Different Basis Sets for the PBE-QIDH­(-D4) and r^2^SCAN-QIDH­(-D4) Models

functional	basis set	MSD	MAD	RMSD	MAX
PBE-QIDH	def2-TZVP	–2.13	2.38	2.48	3.91
	def2-TZVPP	–2.11	2.36	2.46	3.89
	def2-TZVPPD	–2.01	2.29	2.39	3.82
	def2-QZVPPD	–1.72	2.05	2.16	3.61
PBE-QIDH-D4	def2-TZVP	–2.11	2.39	2.49	3.90
	def2-TZVPP	–2.10	2.37	2.47	3.88
	def2-TZVPPD	–1.99	2.30	2.40	3.83
	def2-QZVPPD	–1.70	2.06	2.17	3.60
r^2^SCAN-QIDH	def2-TZVP	–5.74	5.74	5.85	7.09
	def2-TZVPP	–5.73	5.73	5.83	7.02
	def2-TZVPPD	–5.61	5.61	5.72	6.93
	def2-QZVPPD	–5.33	5.33	5.45	6.65
r^2^SCAN-QIDH-D4	def2-TZVP	–5.74	5.74	5.84	7.09
	def2-TZVPP	–5.72	5.72	5.83	7.01
	def2-TZVPPD	–5.61	5.61	5.72	6.92
	def2-QZVPPD	–5.33	5.33	5.45	6.65

### 3.2 Rationalization of the Results for the BLYP- and PBE-Based Family of Functionals

First of all, we will concentrate in the following on the BLYP- and PBE-based family of functionals, seeing their superior performance with respect to the r^2^SCAN-based counterparts, and will do a simple exercise as a proof-of-concept using the experimental Δ*E*_ST_ value^61^ (9.05 kcal/mol) for the simplest carbene, methylene. This value was earlier used by Schaefer et al. to correct the DFT-based calculations of some arylcarbenes^25^ in the form

2 $$\Delta E_{ST}^{corrected}=\Delta E_{ST}-\left(\Delta E_{ST}^{{CH}_{2}}+9.05\right)$$

Therefore, using this crude but effective approximation, one can estimate the initial bias of DFT calculations for overstabilizing the low- or high-spin state of carbenes: BLYP/def2-QZVPPD (PBE/def2-QZVPPD) gives a value of −10.0 (−15.6) kcal/mol for the $\Delta E_{ST}^{{CH}_{2}}$ , already showing the overstabilization of the triplet state by PBE alone, in line with the trends disclosed before. If the correction given by eq 2 is applied to the whole AC12 data set, the error metrics are slightly improved for BLYP (i.e., new MAD and RSMD values of 1.20 and 1.39 kcal/mol, instead of the original 1.27 and 1.58 kcal/mol values) but significantly altered for PBE now (i.e., new MAD and RSMD values of 2.35 and 2.71 kcal/mol, instead of the original 4.12 and 4.36 kcal/mol values). Additionally, seeing the marked interplay between the nature of the exchange–correlation functionals and the weights of their EXX (*a*_*x*_) and PT2 (*a*_*c*_) terms, we will try to disentangle in the following their different influences on the results (the performance of double-hybrid functionals is intriguingly related to these factors).

#### 3.2.1 Comparison of Results for Semilocal and Hybrid Functionals of the BLYP-Based Family: the Role of *a*_*x*_

The need to go beyond semilocal functionals (i.e., those with *a*_*x*_ = 0) might be questioned after looking (Table 2) at the performance of BLYP compared with that of the rest of functionals. The good results of BLYP can also be compared with those provided by the semilocal functional M06-L-D3­(BJ)/def2-TZVPP and its larger MAD value of 5.2 kcal/mol (taken directly from ref 26). Actually, to further isolate the ingredient leading to that accurate performance of BLYP, we also recover the BP86-D3­(BJ)/def2-TZVPP results from ref 18, where a MSD value as high as −4.02 kcal/mol was obtained, and thus truly comparable to values of other semilocal functionals such as PBE-D4, see Table 2. This actually confirms the use of the LYP correlation functional as one of the reasons for the ultimate performance of BLYP-based models, which might be attributed to the self-interaction-free nature of the LYP correlation functional.

However, one should notice that (i) the MSD of BLYP is the lowest among the set of all BLYP-based functionals, thus indicating a marked compensation between the under- and overestimation of values (e.g., the Δ*E*_ST_ value for compound **8** is underestimated by 3.4 kcal/mol while the corresponding value for compound **5** is overestimated by 1.1 kcal/mol); (ii) the MAX error found for this functional (3.4 kcal/mol) is much higher than the MAD value, which is not so markedly observed for other members of the family of BLYP-based functionals neither for any of the PBE-based expressions; and (iii) previous applications of BLYP to the geometry and energy difference between singlet and triplet methylene,^26^ actually the simplest existing carbene, led to a pronounced (marginal) deviation of the bond angle for the singlet (triplet) state, thus indicating an unbalanced treatment of both electronic states.

We would also like to underline next some of the discrepancies and inconsistencies detected along the study by comparing the error metrics provided by semilocal and hybrid functionals. The MAD of B3LYP (*a*_*x*_ = 0.2, MAD = 2.4 kcal/mol) is larger than that of BLYP (*a*_*x*_ = 0, MAD = 1.5 kcal/mol). On the other hand, our own M06–2X/def2-QZVPP calculations (with M06–2X^62^ holding a larger value of *a*_*x*_ = 0.54) led to a MAD of 3.5 kcal/mol, which is now lower than that provided by the M06-L-D3­(BJ)/def2-TZVPP calculations. However, this difference between M06–2X and M06-L might be also attributed to the different parametrization carried out for both M06-L and M06–2X models^63^ and it is thus not conclusive. It thus seems that applying hybrid density functionals with a larger amount (i.e., a higher value of *a*_*x*_) of exact-like exchange does not solve the problem, providing contradictory results (larger or smaller than the corresponding semilocal functionals) depending on the expressions finally used. The application (see Table 4) of MP2 and its spin-component-scaled (SCS-) version (SCS-MP2) is neither the solution, and these calculations are also done here with the large def2-QZVPP basis set, resulting in catastrophic errors as large as 30 kcal/mol.

**Table 4:** MSD, MAD, RMSD, and MAX Errors (kcal/mol) for the AC12 Dataset with Some ad hoc Models^a^

functional	MSD	MAD	RMSD	MAX
MP2	27.63	27.63	29.73	50.21
SCS-MP2	30.21	30.21	31.85	49.68
BLYP-QIDH	1.05	1.24	1.57	3.98
r^2^SCAN2-Pr^2^SCAN	–5.82	5.82	5.91	7.15

a All calculations are done with the def2-QZVPPD basis set.

#### 3.2.2 The Interplay between *a*_*x*_ and *a*_*c*_: Nonempirical Double-Hybrid Functionals

Note that the use of nonempirical functionals allows us to disentangle the effects contributing the most to the results found, contrary to what happens with parametrized models. Therefore, the larger MAD of PBE0 (*a*_*x*_ = 1/4) with respect to PBE (*a*_*x*_ = 0), 6.6 vs 4.4 kcal/mol respectively, can be exclusively attributed to the EXX energy introduced into the former, with a deterioration in MAD of roughly 1 kcal/mol for each increase of a 10% for *a*_*x*_. This rule-of-thumb is corroborated by the comparison between TPSSh-D3­(BJ) (MSD = −5.9 kcal/mol) and TPSS-D3­(BJ) (MSD = −5.0 kcal/mol) performance taken from ref 18, for which it is also concluded that a small value of *a*_*x*_ = 1/10 for the former induced a MAD of almost 1 kcal/mol larger. This dependence of nonempirical models on the *a*_*x*_ weight is also observed by comparing r^2^SCAN and r^2^SCAN0, for which an increase in MAD of 2.2 kcal/mol is also found (see Table 2).

The role played by *a*_*x*_ can also be isolated from the PBE0-DH­(SCF) and PBE-QIDH­(SCF) treatments, where the acronym SCF means Self-Consistent Field and it is thus the result of applying eq 1 neglecting the *a*_*c*_ *E*_*c*_^PT2^ contribution to the electronic energy of double-hybrid functionals. We calculate MAD values of 10.0 and 14.3 kcal/mol for PBE0-DH­(SCF) and PBE-QIDH­(SCF), respectively, thus indicating the need to compensate for those results with an optimal fraction of *a*_*c*_. It is thus confirmed that for nonparameterized hybrid functionals, increasing the *a*_*x*_ values does not help to achieve more accurate results. On the other hand, the r^2^SCAN-QIDH­(SCF) MAD is now 18.2 kcal/mol, and thus higher than that of PBE-QIDH­(SCF) just as a consequence of using the r^2^SCAN instead of the PBE underlying exchange–correlation functional.

Therefore, the only way to improve the results for nonparameterized functionals is by resorting to double-hybrid density functionals as a compromise between robustness, accuracy, and computational cost. Their nonempirical formulation (where *a*_*c*_ = *a*_*x*_^3^ for all PBE0-DH, PBE-QIDH, and r^2^SCAN-QIDH) affords the possibility to use large fractions of exact-like exchange without the penalty and associated error described before, thanks to the balanced introduction of the MP2-like correlation energy. Once the key influence of both factors, *a*_*x*_ and *a*_*c*_ is determined, we can now better compare semiempirical (B2GP-PLYP) and nonempirical (PBE-QIDH) models with close enough *a*_*x*_ and *a*_*c*_ values: PBE-QIDH behaves better than B2GP-PLYP with lower MAD and MAX errors (2.2 and 3.6 kcal/mol vs 2.6 and 5.6 kcal/mol, respectively).

The individual (and signed) deviation for any of the AC12 systems is graphically represented in Figures 2 and 3 for the BLYP-based and PBE-based family of functionals, respectively. Interestingly, the PBE functional is largely biased to favor the triplet state, providing too negative Δ*E*_ST_ values, which is not reduced upon extension to the hybrid PBE0 functional and only partially improves for the PBE0-DH double-hybrid functional. However, PBE-QIDH significantly reduces those signed deviations for every of the systems tackled, bringing the results much closer to the B2-PLYP and B2GP-PLYP values, which was not the case comparing PBE and BLYP or PBE0 and B3LYP.

**Figure 2:**
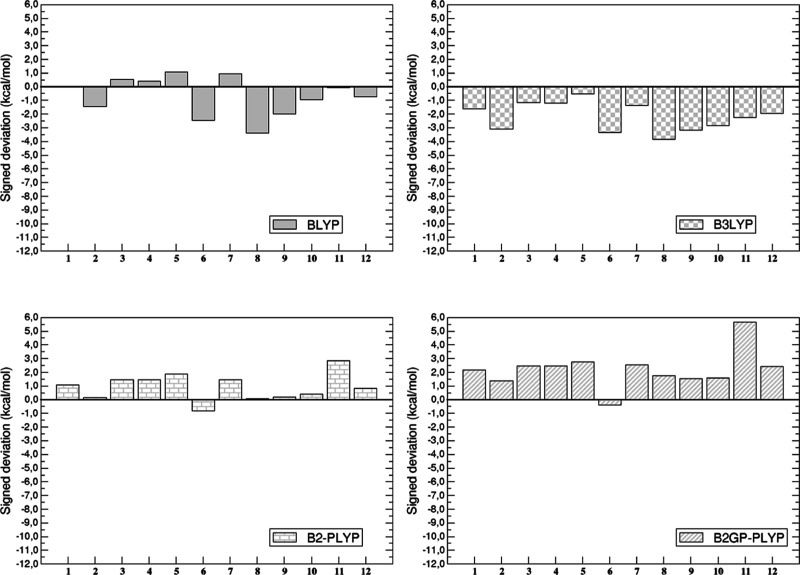
Signed deviation (in kcal/mol) between BLYP-based calculated and reference values for each of the systems of the AC12 data set.

**Figure 3:**
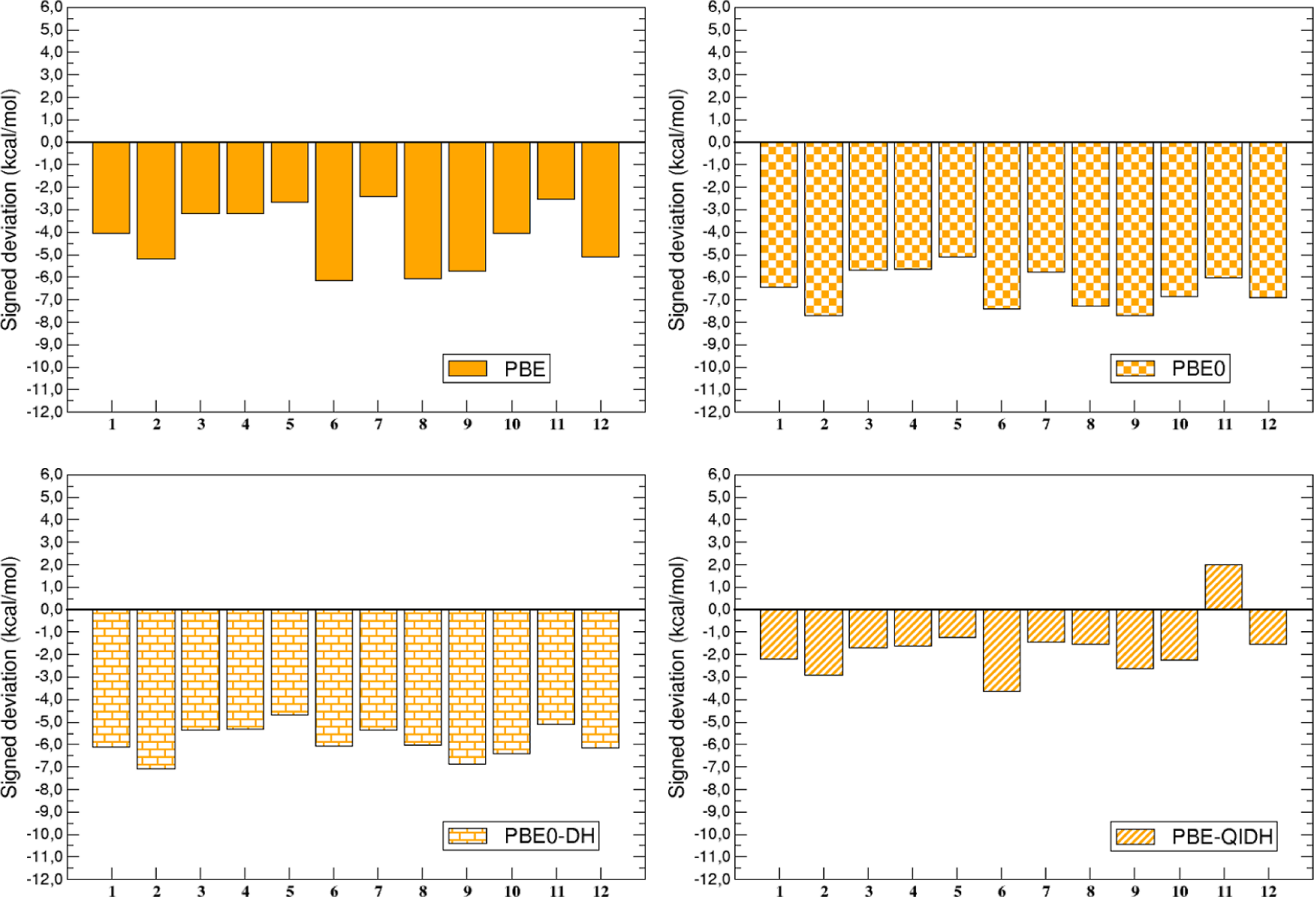
Signed deviation (in kcal/mol) between PBE-based calculated and reference values for each of the systems of the AC12 data set.

The spin contamination values [i.e., deviation for the ideal *S*(*S* + 1) value for the triplet state] are also given as part of the Supporting Information. r^2^SCAN and PBE have a slightly larger spin contamination than BLYP, and the same applies to their hybrid versions, although for hybrid functionals, this is markedly influenced by the EXX weight (*a*_*x*_). The double-hybrid functionals are more influenced by this issue as a consequence of the high spin contamination of their wave function-based terms, with values for PBE-QIDH or r^2^SCAN-QIDH slightly larger than those found for B2-PLYP or B2GP-PLYP due to their different *a*_*x*_ and *a*_*c*_ weights.

Finally, we can now define ad hoc double-hybrid functionals such as BLYP-QIDH (i.e., holding the same values of *a*_*x*_ and *a*_*c*_ than for PBE-QIDH, but using the BLYP instead of the original PBE functional for the *E*_*x*_[ρ] and *E*_*c*_[ρ] terms) or r^2^SCAN2-Pr^2^SCAN (i.e., holding the same values of *a*_*x*_ and *a*_*c*_ than for B2-PLYP, but using the r^2^SCAN instead of the original BLYP functional for the *E*_*x*_[ρ] and *E*_*c*_[ρ] terms). The results collected in Table 4 shows again the marked influence of using r^2^SCAN vs BLYP as the underlying exchange–correlation functional (comparing the new r^2^SCAN2-Pr^2^SCAN vs B2-PLYP) giving rise to larger error for the former, but a slightly less marked influence of BLYP vs PBE (comparing the new BLYP-QIDH vs PBE-QIDH) providing now BLYP-QIDH one of the lowest error metrics of all the whole set of double-hybrid functionals assessed here. Overall, it can be seen how the QIDH coefficients (*a*_*x*_ = 3^–1/3^ and *a*_*c*_ = 1/3) provide a successful scheme for robust calculations.

### 3.3 Global Performance of Functionals on the Weakly Bound DPC-X Systems

The Δ*E*_ST_ singlet–triplet energy gaps of DPC interacting with hydrogen (water and methanol) or halogen bond (XCF_3_, X = Cl, Br, I) donors, thus forming a van der Waals complex, will also be investigated (see Figure 4). Whereas DPC (part of the AC12 data set) keeps a triplet ground-state and a Δ*E*_ST_ value of −3.3 kcal/mol, the formation of the weakly bound complexes always increases that value (Δ*E*_ST_ of −2.1 and −0.7 kcal/mol for DPC···ClCF_3_ and DPC···BrCF_3_, respectively) and even forces it to be positive (Δ*E*_ST_ of 2.0, 1.6, and 3.3 kcal/mol for DPC···H_2_O, DPC···CH_3_OH, and DPC···ICF_3_, respectively) according to the reference values obtained in ref 36.

**Figure 4:**
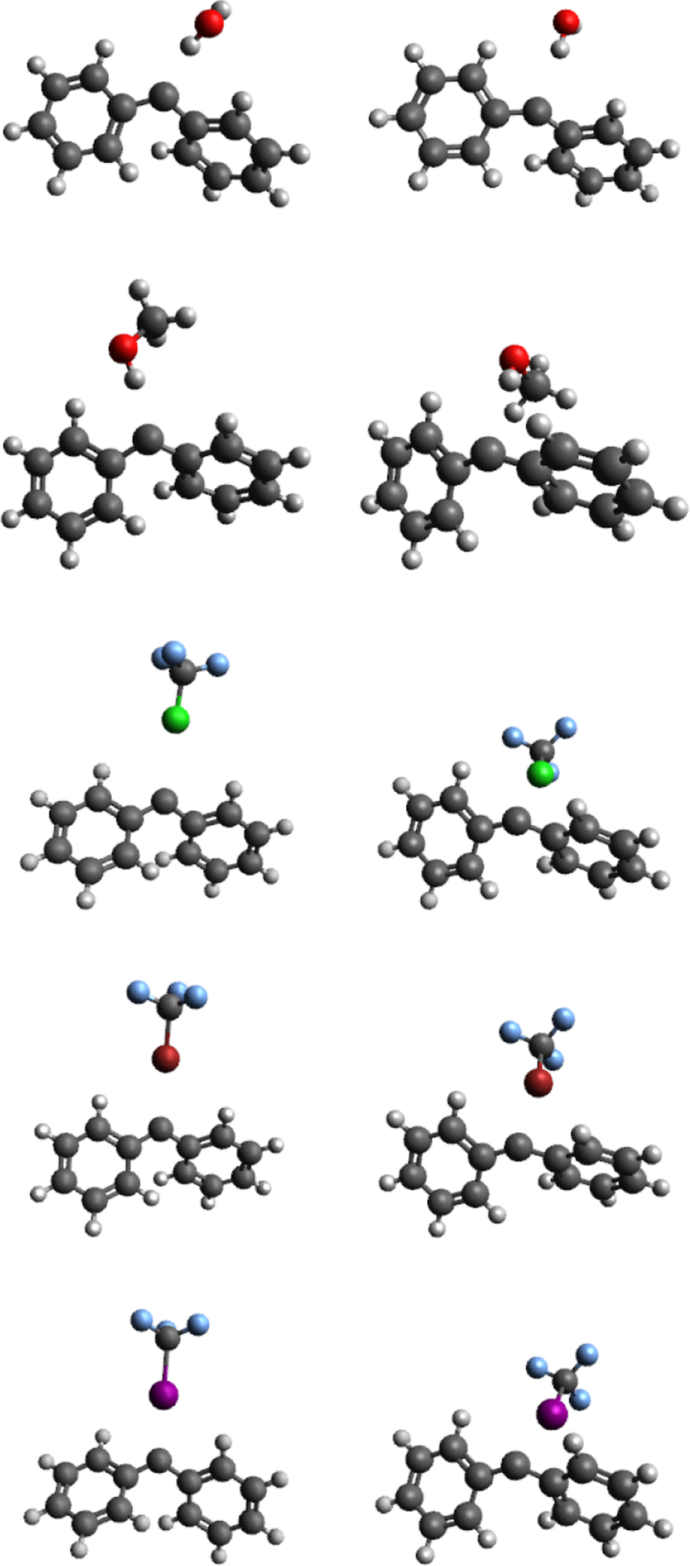
3D-structures of the DPC-based adducts studied, in their singlet (left, ^1^DPC-X) and triplet (right, ^3^DPC-X) states with X = H_2_O, CH_3_OH, ClCF_3_, BrCF_3_, and ICF_3_ molecules (from top to bottom).

Looking first at the performance of the BLYP-based density functionals, see Table 5, B3LYP is remarkably accurate, providing the lowest MAD of around 1 kcal/mol of all the BLYP, B3LYP, B2-PLYP, and B2GP-PLYP functionals, with and without dispersion correction. Actually, the inclusion of the D4 dispersion correction was negligible for double-hybrid density functionals, and impacted negatively for semilocal (BLYP and PBE) or hybrid (B3LYP) functionals in most of the cases. For the family of PBE-based density functionals, the error increases along the hierarchy of methods, similarly to the other family with the exception of B3LYP, but is considerable lower comparing PBE and BLYP, on one hand, and any of the double-hybrid PBE0-DH and PBE-QIDH either with B2-PLYP or B2GP-PLYP. Interestingly, RSX-PBE-QIDH behaves very accurately with a MAD of around 1 kcal/mol thanks to the inclusion of long-range exchange interactions.

**Table 5:** MSD, MAD, RMSD, and MAX Errors (kcal/mol) for the Weakly Bound (DPC-X) Adducts and All the Functionals Investigated^a^

functional	MSD	MAD	RMSD	MAX
BLYP	3.17	3.17	3.74	6.16
BLYP-D4	3.31	3.31	3.94	6.90
B3LYP	0.15	0.92	1.15	1.71
B3LYP-D4	0.26	1.06	1.25	2.33
B2-PLYP	4.63	4.63	4.72	6.16
B2-PLYP-D4	4.64	4.64	4.74	6.38
B2GP-PLYP	7.16	7.16	7.19	8.27
B2GP-PLYP-D4	7.14	7.14	7.17	8.38
PBE	1.09	1.78	2.50	4.89
PBE-D4	1.49	2.20	3.00	5.27
PBE0	–3.42	3.24	3.61	4.87
PBE0-D4	–3.43	3.43	3.64	4.53
PBE0-DH	–3.25	3.25	3.33	4.07
PBE0-DH-D4	–3.28	3.28	3.37	4.12
PBE-QIDH	3.59	3.59	3.64	4.65
PBE-QIDH-D4	3.55	3.55	3.61	4.70
RSX-PBE-QIDH	0.97	0.97	1.06	1.57
r^2^SCAN	–5.31	5.31	5.56	6.98
r2SCAN-D4	–5.36	5.36	5.62	6.83
r^2^SCAN0	–9.62	9.62	9.65	10.44
r2SCAN0-D4	–9.68	9.68	9.71	10.50
r2SCAN-QIDH	–0.96	0.96	1.09	1.64
r2SCAN-QIDH-D4	–0.98	0.98	1.12	1.70
BLYP-QIDH	5.28	5.28	5.30	5.87
r2SCAN2-Pr2SCAN	–1.73	1.73	1.97	2.62

a All calculations are done with the def2-QZVPP basis set.

The case of r^2^SCAN-based functionals will also be analyzed next. The semilocal r^2^SCAN form provides a large MAD compared to other functionals, and the r^2^SCAN0 hybrid expression actually yields the largest error among all the data shown in Table 5. However, r^2^SCAN-QIDH becomes as competitive as RSX-PBE-QIDH, with an MAD of around 1 kcal/mol. Actually, of all the functionals considered, only RSX-PBE-QIDH and r^2^SCAN-QIDH are able to provide those low errors together with the right sign of the Δ*E*_ST_ singlet–triplet energy gaps for all of the DPC···H_2_O, DPC···CH_3_OH, DPC···ClCF_3_, DPC···BrCF_3_, and DPC···ICF_3_ systems.

The use of the BLYP exchange–correlation functional into the QIDH scheme (i.e., BLYP-QIDH) did not bring any additional advantage, actually inheriting the worse performance of BLYP vs PBE commented on above. On the other hand, introducing the r^2^SCAN exchange–correlation functional into the B2-PLYP scheme (i.e., r^2^SCAN2-Pr^2^SCAN) substantially improved the B2-PLYP results, reducing the MAD of 4.6 to 1.7 kcal/mol, showing again the subtle interplay between the ingredients defining a double-hybrid density functional.

## 4 Conclusions

We have used the AC12 set of arylcarbenes and the DPC···X set of weakly interacting systems, for which nearly exact DLPNO–CCSD­(T)/CBS results for their singlet–triplet energy gaps were available, to assess the accuracy of a wide and representative range of density functionals expressions (semilocal, hybrid, and double-hybrid functionals). We have selected two families of methods: (i) semiempirical BLYP, B3LYP, B2-PLYP, and B2GP-PLYP and (ii) nonempirical PBE, PBE0, PBE0-DH, and (RSX-)­PBE-QIDH, as well as their extensions to r^2^SCAN, r^2^SCAN0, and r^2^SCAN-QIDH. This representation of functionals have allowed to disentangle the role played by (i) the underlying exchange–correlation functional (i.e., BLYP, PBE or r^2^SCAN) and (ii) the specific weights given to the EXX contribution (hybrid) and to the EXX and the second-order perturbation theory correlation energy contributions (double-hybrid). The results revealed a delicate but marked interplay among the factors described above.

First of all, before isolating other factors contributing to the overall performance of the methods, all of the results are obtained at the converged def2-QZVPPD basis set, thus avoiding basis set incompleteness or superposition errors (e.g., for the DPC···X set of weakly interacting systems). Furthermore, all the methods were coupled with the D4 correction for intra- and intermolecular noncovalent effects, which shows a negligible impact on the results for double-hybrid methods and, more generally, a slight deterioration of the results for some of the rest of the functionals.

For the AC12 data set, PBE leads to a marked overstabilization of the triplet state, inherited by the poor description of the methylene (simplest) carbene, and thus to too negative singlet–triplet energy differences contrarily to BLYP results. However, the PBE-QIDH model is able to remarkably compensate for that initial overstabilization, competing in accuracy with other double-hybrid methods like, e.g., B2GP-PLYP. The use of the r^2^SCAN exchange–correlation functional showed to be detrimental, be it alone or forming the corresponding r^2^SCAN-QIDH double-hybrid functional. Overall, a MAD of 1 (2) kcal/mol is only achieved by BLYP or B2-PLYP (B3LYP, B2GP-PLYP, or PBE-QIDH). For the DPC···X data set of weakly interacting systems, the results are dominated by the capacity of the model to introduce medium- to long-range energy contributions, with RSX-PBE-QIDH and r^2^SCAN-QIDH providing now MAD of 1 kcal/mol, closely followed only by B3LYP.

The double-hybrid B2-PLYP and B2GP-PLYP functionals behave very differently for both data sets, with a 3- or 4-fold increase of the MAD values going from the AC12 (values of 1–2 kcal/mol, roughly speaking) to the DPC···X data set (values of 5–7 kcal/mol, roughly speaking). On the other hand, PBE-QIDH does not show the best performance among the models assessed for any of both data sets, but it keeps instead a more homogeneous performance with MAD between 2–4 kcal/mol. The RSX-PBE-QIDH extension also reasonably performs for both data sets with MAD between 1 and 3 kcal/mol. This different and subtle performance of the models on the two data sets pinpoints to the intrinsic difficulty of automated or machine-learned models to cope with the electronic effects driving the results and to the careful selection of models for energy difference involving carbene systems.

## Supplementary Material



## Data Availability

The data that supports the findings of this study are available within the article [and its Supporting Information] or are available from the corresponding authors upon reasonable request.

## Notes

Individual values for Δ*E*_ST_ values calculated by all functionals with the def2-QZVPPD basis set for each of the system of the AC12 data set; Δ*E*_ST_ values calculated by double-hybrid functionals and a variety of increasingly larger basis sets for each of the systems of the AC12 data set; spin contamination by all functionals for each of the system of the AC12 data set; signed deviation between r^2^SCAN-based calculated and reference values for each of the systems of the AC12 data set; Δ*E*_ST_ values calculated by all functionals with the def2-QZVPP­(D) basis set for each of the weakly bound (DPC-X) systems; and spin contamination by all functionals for each of the weakly bound (DPC-X) systems (PDF)
