# Supplementary material for: Singlet–Triplet Gap in Covalently and Weakly Bound Carbenes: Studying the Dependence on the Exchange–Correlation Functional
Source: ACS Omega. 2025 Oct 17;10(42):50371–81. doi: 10.1021/acsomega.5c07611 (PMC12573174; doi:10.1021/acsomega.5c07611)
Supplement: Supplementary file 1 [file ao5c07611_si_001.pdf]

Singlet-Triplet Gap in Covalently and Weakly Bound Carbenes: Studying the Dependence on the Exchange-Correlation Functional.

Pablo Maiz-Pastor[a], Éric Brémond[b], Ángel José Pérez-Jiménez[a], Carlo Adamo[c], and Juan Carlos Sancho-García[a*]

[a] Department of Physical Chemistry,
University of Alicante,
E-03080 Alicante, Spain

[b] Université de Paris, ITODYS, CNRS,
F-75006 Paris, France

[c] Chimie ParisTech, PSL Research University, CNRS,
Institute of Chemistry for Life and Health Sciences (i-CLeHS), FRE 2027,
F-75005 Paris, France

October 2, 2025

*E-mail: jc.sancho@ua.es

Table S1. Singlet-triplet energy differece (in kcal/mol) of the AC12 dataset for the functionals considered. All calculations are done with the def2-QZVPPD basis set.

Functional	**1**	**2**	**3**	**4**	**5**	**6**	**7**	**8**	**9**	**10**	**11**	**12**
BLYP	–4.08	–9.34	0.44	0.11	2.41	20.08	–3.72	21.67	15.07	6.70	–3.40	–3.58
BLYP-D4	–3.98	–9.26	0.55	0.23	2.53	20.21	–3.62	21.32	15.13	6.95	–2.35	–3.56
B3LYP	–5.69	–10.98	–1.22	–1.48	0.83	19.21	–6.04	21.19	13.90	4.83	–5.61	–4.79
B3LYP-D4	–5.65	–10.96	–1.17	–1.42	0.90	19.29	–6.01	20.99	13.97	5.04	–4.78	–4.78
B2-PLYP	–2.98	–7.74	1.39	1.14	3.21	21.72	–3.21	25.13	17.25	8.04	–0.51	–2.03
B2-PLYP-D4	–2.97	–7.73	1.41	1.16	3.25	21.75	–3.19	25.02	17.27	8.13	–0.12	–2.03
B2GP-PLYP	–1.90	–6.52	2.38	2.17	4.11	22.16	–2.10	26.79	18.57	9.24	2.28	–0.42
B2GP-PLYP-D4	–1.89	–6.51	2.39	2.18	4.13	22.18	–2.09	26.72	18.58	9.29	2.53	–0.41
Reference	–4.06	–7.89	–0.08	–0.30	1.35	22.54	–4.66	25.05	17.05	7.65	–3.36	–2.84

Table S2. Singlet-triplet energy differece (in kcal/mol) of the AC12 dataset for the functionals considered. All calculations are done with the def2-QZVPPD basis set.

Functional	**1**	**2**	**3**	**4**	**5**	**6**	**7**	**8**	**9**	**10**	**11**	**12**
PBE	–8.11	–13.06	–3.23	–3.47	–1.33	16.39	–7.07	18.98	11.33	3.58	–5.88	–7.95
PBE-D4	–8.09	–13.04	–3.19	–3.43	–1.28	16.44	–7.04	18.85	11.37	3.70	–5.35	–7.94
PBE0	–10.52	–15.58	–5.77	–5.94	–3.77	15.12	–10.45	17.75	9.35	0.80	–9.37	–9.76
PBE0-D4	–10.50	–15.56	–5.74	–5.91	–3.73	15.16	–10.43	17.62	9.39	0.91	–8.89	–9.75
PBE0-DH	–10.15	–14.98	–5.45	–5.61	–3.33	16.48	–10.01	19.04	10.19	1.25	–8.44	–8.99
PBE0-DH-D4	–10.15	–14.97	–5.45	–5.60	–3.32	16.49	–10.00	18.99	10.20	1.28	–8.28	–8.98
PBE-QIDH	–6.26	–10.81	–1.78	–1.92	0.12	18.93	–6.12	23.53	14.43	5.40	–1.35	–4.36
PBE-QIDH-D4	–6.25	–10.81	–1.77	–1.91	0.13	18.94	–6.12	23.49	14.44	5.43	–1.19	–4.35
RSX-PBE-QIDH	–8.17	–12.71	–3.60	–3.71	–1.63	17.90	–8.09	22.58	12.92	3.33	–3.74	–5.76
Reference	–4.06	–7.89	–0.08	–0.30	1.35	22.54	–4.66	25.05	17.05	7.65	–3.36	–2.84

Table S3. Singlet-triplet energy differece (in kcal/mol) of the AC12 dataset for the functionals considered. All calculations are done with the def2-QZVPPD basis set.

Functional	**1**	**2**	**3**	**4**	**5**	**6**	**7**	**8**	**9**	**10**	**11**	**12**
r^2SCAN	–13.96	–18.89	–8.64	–8.89	–6.51	12.70	–12.54	14.50	6.03	–1.70	–10.87	–12.71
r^2SCAN-D4	–13.95	–18.89	–8.64	–8.88	–6.50	12.71	–12.54	14.45	6.04	–1.68	–10.68	–12.71
r^2SCAN0	–16.09	–21.06	–10.98	–11.17	–8.81	11.42	–15.60	12.95	4.02	–4.42	–14.59	–14.51
r^2SCAN0-D4	–16.09	–21.05	–10.97	–11.16	–8.80	11.43	–15.59	12.89	4.03	–4.39	–14.38	–14.51
r^2SCAN-QIDH	–10.05	–14.54	–5.37	–5.55	–3.41	16.24	–9.85	19.94	10.65	1.74	–5.55	–7.82
r^2SCAN-QIDH-D4	–10.05	–14.54	–5.37	–5.55	–3.41	16.25	–9.85	19.93	10.66	1.75	–5.50	–7.82
MP2	18.04	37.89	21.75	21.55	22.74	35.23	18.83	49.60	39.85	31.50	46.85	38.18
SCS-MP2	21.17	40.63	24.85	24.68	25.84	39.87	21.80	51.62	42.51	33.50	46.32	40.14
BLYP-QIDH	–3.05	–7.72	1.26	1.07	3.02	21.40	–3.43	25.76	17.43	7.88	0.62	–1.20
r^2SCAN2-Pr^{2}SCAN	–10.49	–15.04	–5.70	–5.91	–3.65	16.12	–9.95	19.14	10.09	1.52	–6.54	–9.00
Reference	–4.06	–7.89	–0.08	–0.30	1.35	22.54	–4.66	25.05	17.05	7.65	–3.36	–2.84

Table S4. Singlet-triplet energy differece (in kcal/mol) of the AC12 dataset for the functionals and basis sets considered.

Functional	**1**	**2**	**3**	**4**	**5**	**6**	**7**	**8**	**9**	**10**	**11**	**12**
PBE-QIDH/def2-TZVP	–6.75	–11.26	–2.29	–2.37	–0.33	18.63	–6.53	23.21	14.12	5.03	–1.85	–4.71
PBE-QIDH/def2-TZVPP	–6.70	–11.18	–2.24	–2.34	–0.34	18.65	–6.44	23.20	14.09	5.02	–1.86	–4.76
PBE-QIDH/def2-TZVPPD	–6.55	–11.10	–2.07	–2.23	–0.19	18.71	–6.48	23.25	14.13	5.10	–1.66	–4.57
PBE-QIDH/def2-QZVPPD	–6.26	–10.81	–1.78	–1.92	0.12	18.93	–6.12	23.53	14.43	5.40	–1.35	–4.36
PBE-QIDH-D4/def2-TZVP	–6.75	–11.26	–2.28	–2.37	–0.32	18.64	–6.53	23.17	14.13	5.06	–1.69	–4.71
PBE-QIDH-D4/def2-TZVPP	–6.69	–11.18	–2.24	–2.33	–0.33	18.66	–6.44	23.16	14.10	5.05	–1.70	–4.75
PBE-QIDH-D4/def2-TZVPPD	–6.54	–11.10	–2.06	–2.22	–0.18	18.72	–6.48	23.20	14.14	5.13	–1.49	–4.57
PBE-QIDH-D4/def2-QZVPPD	–6.25	–10.81	–1.77	–1.91	0.13	18.94	–6.12	23.49	14.44	5.43	–1.19	–4.35
r^2SCAN-QIDH/def2-TZVP	–10.54	–14.98	–5.88	–6.00	–3.84	15.92	–10.26	19.62	10.35	1.37	–6.06	–8.17
r^2SCAN-QIDH/def2-TZVPP	–10.49	–14.91	–5.84	–5.97	–3.86	15.94	–10.17	19.60	10.33	1.36	–6.06	–8.21
r^2SCAN-QIDH/def2-TZVPPD	–10.33	–14.82	–5.65	–5.84	–3.71	16.02	–10.20	19.66	10.37	1.44	–5.84	–8.01
r^2SCAN-QIDH/def2-QZVPPD	–10.05	–14.54	–5.37	–5.55	–3.41	16.24	–9.85	19.94	10.65	1.74	–5.55	–7.82
r^2SCAN-QIDH-D4/def2-TZVP	–10.54	–14.98	–5.88	–5.99	–3.84	15.92	–10.26	19.60	10.35	1.37	–6.01	–8.17
r^2SCAN-QIDH-D4/def2-TZVPP	–10.49	–14.90	–5.84	–5.96	–3.86	15.94	–10.17	19.58	10.33	1.36	–6.01	–8.21
r^2SCAN-QIDH-D4/def2-TZVPPD	–10.32	–14.81	–5.65	–5.84	–3.71	16.03	–10.20	19.64	10.37	1.45	–5.79	–8.01
r^2SCAN-QIDH-D4/def2-QZVPPD	–10.05	–14.54	–5.37	–5.55	–3.41	16.25	–9.85	19.93	10.66	1.75	–5.50	–7.82
B2-PLYP/def2-TZVPP	–3.38	–8.02	0.95	0.77	2.80	21.45	–3.44	24.83	16.97	7.73	–0.95	–2.43
B2-PLYP-D4/def2-TZVPP	–3.37	–8.01	0.97	0.80	2.83	21.48	–3.42	24.73	17.00	7.81	–0.56	–2.42
B2-PLYP/def2-QZVPPD	–2.98	–7.74	1.39	1.14	3.21	21.72	–3.21	25.13	17.25	8.04	–0.51	–2.03
B2-PLYP-D4/def2-QZVPPD	–2.97	–7.73	1.41	1.16	3.25	21.75	–3.19	25.02	17.27	8.13	–0.12	–2.03

Table S5. Spin contamination (triplet) for the molecules of the AC12 dataset for the functionals considered. All calculations are done with the def2-QZVPPD basis set.

Functional	**1**	**2**	**3**	**4**	**5**	**6**	**7**	**8**	**9**	**10**	**11**	**12**
BLYP	2.017	2.017	2.016	2.016	2.016	2.010	2.017	2.013	2.014	2.014	2.021	2.012
B3LYP	2.038	2.040	2.037	2.037	2.037	2.025	2.040	2.032	2.034	2.033	2.053	2.029
B2-PLYP	2.142	2.146	2.132	2.138	2.136	2.076	2.153	2.127	2.133	2.131	2.229	2.123
B2GP-PLYP	2.212	2.217	2.202	2.207	2.202	2.107	2.229	2.195	2.202	2.200	2.364	2.204
PBE	2.020	2.020	2.018	2.019	2.019	2.012	2.019	2.015	2.016	2.016	2.024	2.015
PBE0	2.054	2.057	2.051	2.053	2.052	2.034	2.054	2.045	2.048	2.047	2.077	2.041
PBE0-DH	2.134	2.138	2.127	2.130	2.127	2.073	2.138	2.119	2.124	2.123	2.213	2.114
PBE-QIDH	2.257	2.263	2.243	2.249	2.243	2.127	2.267	2.237	2.245	2.244	2.451	2.260
RSX-PBE-QIDH	2.298	2.306	2.284	2.290	2.285	2.154	2.309	2.278	2.286	2.287	2.539	2.328
r^2SCAN	2.042	2.042	2.038	2.038	2.038	2.020	2.037	2.034	2.036	2.036	2.057	2.031
r^2SCAN0	2.081	2.083	2.075	2.077	2.076	2.043	2.077	2.070	2.073	2.072	2.121	2.064
r^2SCAN-QIDH	2.270	2.273	2.257	2.262	2.257	2.133	2.279	2.249	2.258	2.258	2.480	2.280
(SCS-)MP2	2.610	3.064	2.592	2.601	2.592	2.369	2.630	2.590	2.601	2.610	3.200	2.951
BLYP-QIDH	2.234	2.240	2.224	2.229	2.224	2.118	2.253	2.217	2.225	2.223	2.410	2.234
r^2SCAN2-Pr^2SCAN	2.191	2.193	2.179	2.183	2.180	2.094	2.191	2.172	2.179	2.178	2.320	2.175

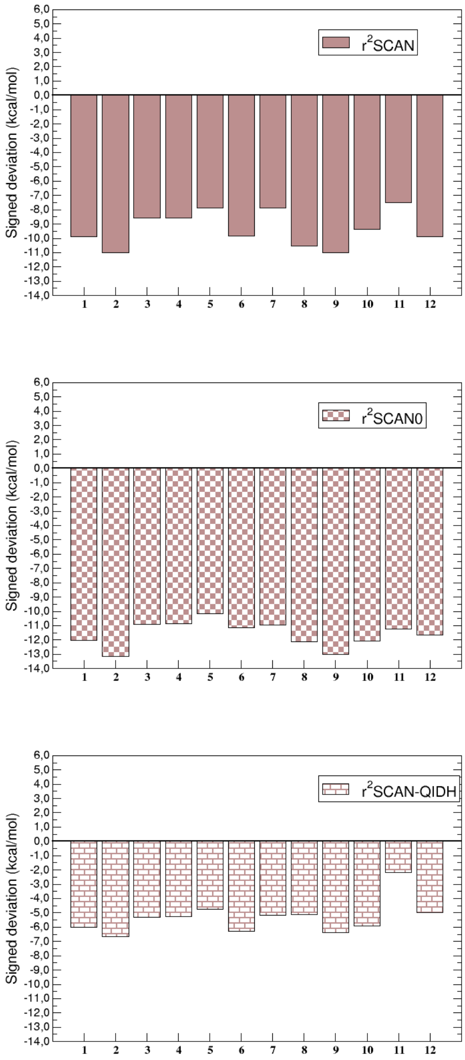

Figure S1. Signed deviation (in kcal/mol) between r^{2}SCAN-based calculated and reference values for each of the systems of the AC12 dataset

Table S6. Singlet-triplet energy differece (in kcal/mol) of the weakly bound (DPC-X) adducts for the functionals considered. All calculations are done with the def2-QZVPP basis set.

Functional	DPC-H_2O	DPC-CH_3OH	DPC-$ClCF_3$	DPC-$BrCF_3$	DPC-ICF_3
BLYP	2.32	4.01	0.43	3.73	9.49
BLYP-D4	3.30	3.16	0.10	3.89	10.23
B3LYP	0.37	1.64	–2.38	0.19	5.04
B3LYP-D4	1.17	1.04	–2.72	0.27	5.66
B2-PLYP	5.52	6.02	1.91	4.34	9.49
B2-PLYP-D4	5.87	5.68	1.73	4.35	9.71
B2GP-PLYP	8.45	8.71	4.48	6.67	11.60
B2GP-PLYP-D4	8.67	8.45	4.33	6.64	11.71
PBE	0.43	1.67	–2.22	1.46	8.22
PBE-D4	0.93	1.24	–2.45	3.27	8.60
PBE0	–2.84	–1.92	–6.36	–3.66	1.81
PBE0-D4	–2.40	–2.37	–6.63	–3.69	2.07
PBE0-DH	–2.04	–1.51	–6.08	–3.81	1.32
PBE0-DH-D4	–1.90	–1.69	–6.22	–3.85	1.37
PBE-QIDH	5.16	5.32	0.73	2.86	7.98
PBE-QIDH-D4	5.30	5.14	0.60	2.82	8.03
RSX-PBE-QIDH	3.18	3.14	–1.78	–0.02	4.46
r^2SCAN	–4.95	–4.55	–8.48	–5.41	0.96
r^2SCAN-D4	–4.80	–4.77	–8.65	–5.46	1.02
r^2SCAN0	–8.41	–8.09	–12.41	–10.13	–4.93
r^2SCAN0-D4	–8.24	–8.34	–12.60	–10.20	–4.87
r^2SCAN-QIDH	0.81	0.71	–3.74	–1.72	3.28
r^2SCAN-QIDH-D4	0.85	0.64	–3.80	–1.74	3.30
BLYP-QIDH	6.84	7.05	2.77	4.69	9.20
r^2SCAN2-Pr^2SCAN	–0.39	–0.43	–4.72	–2.30	3.33
Reference	2.03	1.57	–2.10	–0.70	3.33

Table S7. Spin contamination (triplet) of the weakly bound (DPC-X) adducts for the functionals considered. All calculations are done with the def2-QZVPPD basis set.

Functional	DPC-H_2O	DPC-CH_3OH	DPC-$ClCF_3$	DPC-$BrCF_3$	DPC-ICF_3
BLYP	2.020	2.020	2.020	2.020	2.020
B3LYP	2.051	2.052	2.051	2.050	2.050
B2-PLYP	2.227	2.228	2.224	2.225	2.225
B2GP-PLYP	2.366	2.366	2.363	2.363	2.363
PBE	2.023	2.024	2.023	2.023	2.023
PBE0	2.074	2.075	2.073	2.073	2.073
PBE0-DH	2.211	2.212	2.208	2.208	2.208
PBE-QIDH	2.456	2.453	2.453	2.453	2.453
RSX-PBE-QIDH	2.546	2.546	2.543	2.543	2.543
r^2SCAN	2.055	2.055	2.054	2.054	2.054
r^2SCAN0	2.118	2.118	2.116	2.116	2.116
r^2SCAN-QIDH	2.485	2.484	2.481	2.481	2.481
BLYP-QIDH	2.413	2.413	2.410	2.410	2.410
r^2SCAN2-Pr^2SCAN	2.321	2.320	2.317	2.317	2.317
